# Imaging findings of acute calcific periarthritis, with emphasis on magnetic resonance imaging: pictorial essay

**DOI:** 10.1590/0100-3984.2023.0126-en

**Published:** 2024-05-07

**Authors:** Letícia Bianco Gomes de Almeida, Marcelo Mantiolhe Martins, Vinícius Neves Marcos

**Affiliations:** 1 Department of Diagnostic Imaging, Hospital Universitário da Universidade Federal de Juiz de Fora/Empresa Brasileira de Serviços Hospitalares (HU-UFJF/EBSERH), Juiz de Fora, MG, Brazil

**Keywords:** Periarthritis, Joint diseases, Calcinosis, Hydroxyapatites, Magnetic resonance imaging, Diagnostic imaging, Periartrite, Artropatia por cristais, Calcinose, Hidroxiapatitas, Ressonância magnética, Diagnóstico por imagem

## Abstract

Acute calcific periarthritis (ACP) is defined as periarticular inflammation associated with intra-articular deposits of hydroxyapatite and other basic calcium phosphate crystals. Patients with ACP present with a sudden onset of pain, together with localized swelling, as well as erythema, tenderness, and reduced range of motion. Familiarity with the clinical and radiological manifestations of ACP aids in the diagnosis and helps differentiate it from other conditions, particularly infectious or inflammatory pathologies such as septic arthritis and gout, thereby reducing the number of unnecessary diagnostic and therapeutic procedures. The objective of this pictorial essay is to illustrate the imaging findings of ACP in various joints, with an emphasis on the findings obtained by magnetic resonance imaging.

## Abstract (pt)

A periartrite cálcica aguda (PCA) é uma inflamação periarticular aguda associada a depósitos justa-articulares de hidroxiapatita e outros cristais básicos de fosfato de cálcio. Os pacientes apresentam início súbito de dor, edema localizado, eritema, sensibilidade e redução da amplitude de movimentos. A familiaridade com as manifestações clínicas e radiológicas da PCA facilita o diagnóstico e permite diferenciá-la de outras entidades, em particular, com doenças infecciosas ou inflamatórias, como artrite séptica e gota, reduzindo procedimentos diagnósticos e terapêuticos desnecessários. O objetivo deste ensaio iconográfico é ilustrar os achados de imagem de PCA em algumas articulações, com ênfase na ressonância magnética.

## INTRODUCTION

Acute calcium periarthritis (ACP) is defined as acute periarticular inflammation associated with juxta-articular deposits of hydroxyapatite and other basic calcium phosphate crystals. Calcifications can be seen in the cartilage, synovium, capsule, tendons, bursa, ligaments, soft tissues, and blood vessels^(1)^. In the literature, various terms have been used in order to describe periarticular calcium deposits, including calcific tendinopathy, calcific bursitis, and calcific peritendinitis, depending on the structure affected^(2)^.

Although the shoulder is joint most commonly affected by ACP, there have also been reports of ACP of the hand, wrist, hip, thigh, knee, ankle, and foot^(3)^. The etiology and pathophysiology of this condition are not yet fully understood^(4)^. Local factors, such as ischemia or trauma, might play an important role in the initiation of periarticular deposition of calcium crystals^(2)^. The condition affects individuals of all ages, being most common in those between 30 and 60 years of age, and affects both genders, with a slight predilection for females^(5)^.

Because ACP has various clinical presentations, many cases are misdiagnosed as septic arthritis or inflammatory arthritis of another nature, resulting in unnecessary diagnostic procedures, treatments, and hospital admissions, as well as the use of inappropriate medications^(3)^. There are certain associations between ACP and systemic diseases such as rheumatoid arthritis, gout, pseudogout, hypothyroidism, and diabetes mellitus, which can result in diagnostic confusion^(1)^.

The primary objective of this article is to illustrate the imaging findings of ACP in different modalities, with an emphasis on magnetic resonance imaging. We also discuss the aspects that help differentiate ACP from other diseases.

## EVOLUTION, SYMPTOMS AND CLINICAL MANAGEMENT

The evolution of ACP can be divided into four phases. During the first (pre-calcific) phase, there is asymptomatic fibrocartilaginous metaplasia of the tendon fibers. The second (formative) phase is characterized by the formation of calcium crystals, with variable symptoms. The third (resorptive) phase is the most symptomatic and disabling, because of the local inflammatory process caused by crystals being reabsorbed, extravasated into the adjacent tissues, or both. In the fourth (post-calcific) phase, there is tissue repair, including the formation of new capillaries and collagen fibers, with some pain and restricted movement, both of which can last for months^(5)^.

The symptoms most commonly reported by patients with ACP are pain, local edema, erythema, sensitivity, and reduced range of motion^(6)^. Because they are self-limited, these symptoms become less severe within four to seven days after the abrupt onset of pain and resolve within three to four weeks, even without treatment. Recurrence is uncommon^(1,3)^. The symptoms of ACP can be treated with local anesthetics, corticosteroids, oral non-steroidal anti-inflammatory drugs, and immobilization^(1)^. The administration of corticosteroids and local anesthetics can be guided by computed tomography or ultrasound^(7)^.

## IMAGING FINDINGS

On radiographs of individuals with ACP, calcifications are seen as distinct, well-circumscribed, homogeneous, periarticular densities, without internal trabeculae or a definable cortex, and can be located within the joint capsule or within adjacent tendons/peritendinous tissues and ligaments^(6)^. There is no limit to the extent of the calcification, which varies significantly, and the size of the calcification does not correlate with symptom severity^(8)^. Over time, mineralization-related changes in morphology and configuration become less well defined and can result in fragmentation. In most cases, the deposits decrease markedly in size or disappear completely within two or three weeks^(8)^. However, in some cases, they can persist for months^(9)^.

**Figure 1:**
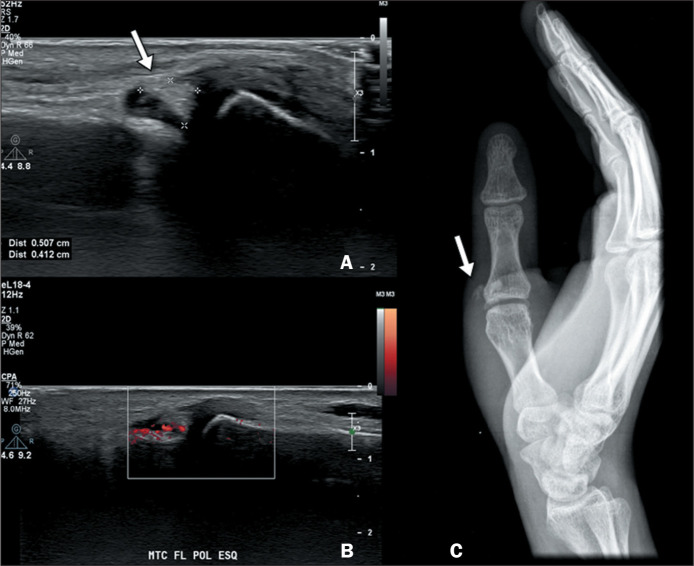
25-year-old male patient with a three-day history of arthralgia in the metacarpophalangeal joint of the left thumb, with edema, hyperemia, and local heat. The patient had no history of trauma. Because local arthritis was suspected, an ultrasound examination was ordered. A,B: Ultrasound images showing a periarticular echogenic focus (arrow) near the radial collateral ligament of the metacarpophalangeal joint of the thumb, without posterior acoustic shadowing and with increased adjacent vascularization on power Doppler. C: Radiograph taken on the same day, confirming the presence of periarticular calcium deposits (arrow).

Ultrasound is quite useful for ACP in some joints, especially those such as the fingers, wrist, and shoulder, where the calcific deposits are more superficial, manifesting in different ways according to their stage. In the initial (pre-calcific) phase, ultrasound shows hyperechoic foci with well-defined borders and posterior acoustic shadowing. When symptoms intensify, there is fragmentation of the deposits, which begin to have a creamy consistency, similar to toothpaste or milk, being identified as hyperechoic foci with ill-defined borders and often without posterior acoustic shadowing, and can erode the cortical bone or invade the bursae. It is at this moment that the increase in echogenicity of the surrounding tissues and the adjacent capsular and pericapsular hyperemia are seen most clearly on color Doppler (Figure 1). In the final (post-calcific) phase, the deposits appear as small hyperechoic foci with well-defined borders and no posterior acoustic shadowing, accompanied by intratendinous cysts^(7)^.

**Figure 2:**
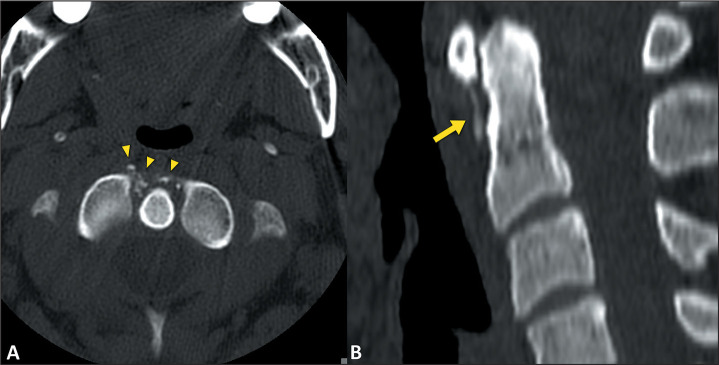
A 47-year-old male patient underwent tomography of the cervical spine, performed for investigation of neck pain and torticollis. Calcifications can be seen anterior to the C2 odontoid process, affecting the area of the anterior longitudinal ligament (arrowheads in A) and extending to the longus colli muscle of the neck (arrow in B).

Computed tomography provides a detailed view of the deposits in ACP. It allows them to be characterized by size, shape, and location, as well as facilitating the identification of high-density zones within the soft tissues, which are often accompanied by increased attenuation in the adjacent soft tissues, indicative of a local inflammatory process^(7)^, as illustrated in Figure 2.

**Figure 3:**
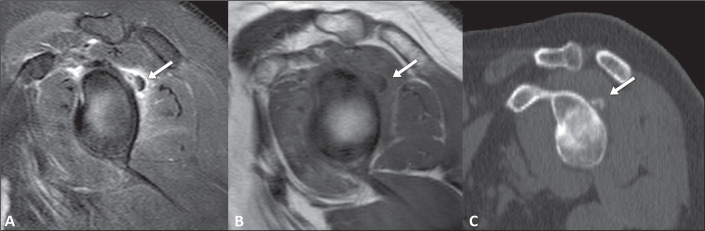
A 56-year-old female patient with a seven-day history of shoulder pain underwent magnetic resonance imaging and subsequent computed tomography. A: Sagittal T2 image with fat saturation showing low signal foci (arrow) in the posterosuperior capsulolabral region of the shoulder, with edema of adjacent soft tissues. B: Sagittal T1-weighted image showing foci with low signal intensity in the posterosuperior capsulolabral region (arrow). C: Sagittal computed tomography reconstruction demonstrating foci of calcification (arrow).

On magnetic resonance imaging, ACP calcifications typically appear as oval, well-defined foci, with low signal intensity on all pulse sequences (Figure 3). There have been reported cases of calcified deposits that, because of their fluid content, are hyperintense on T2-weighted sequences^(4,10)^. Therefore, the calcified lesion may appear hyperintense or hypointense on T2-weighted images, possibly depending on the chemical properties of the crystal, the T2 relaxation time of the underlying inflammatory tissue, the fluid content of the acute lesions, or a combination of those factors^(10)^.

**Figure 4:**
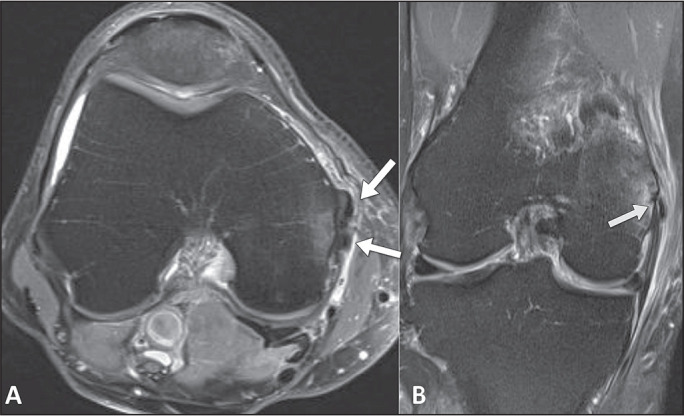
A 44-year-old male patient with a two-day history of pain in the medial aspect of the knee and a history of contusion due to a fall from standing height 20 days prior. A: Axial T2-weighted image with fat saturation, showing foci with low signal intensity (arrows), together with edema in the adjacent soft tissues and a pattern of bone edema in the medial femoral condyle. B: Coronal T2-weighted image with fat saturation, showing a small focus with low signal intensity, related to intraosseous calcific migration, with a pattern of bone edema, in the femoral condyle (arrow).

Soft tissue edema is normally present in acute presentations of ACP, correlating with the clinical symptoms. Although uncommon, bone marrow edema, cortical erosion, and intraosseous extension can occur in patients with periarticular calcification^(9)^, as shown in Figure 4.

## DIFFERENTIAL DIAGNOSIS

The clinical presentation of ACP can mimic that of other conditions, mainly infectious processes, inflammatory processes, and arthropathies, as well as, more rarely, neoplasms. Therefore, there is a high rate of misdiagnosis^(3,6)^.

**Figure 5:**
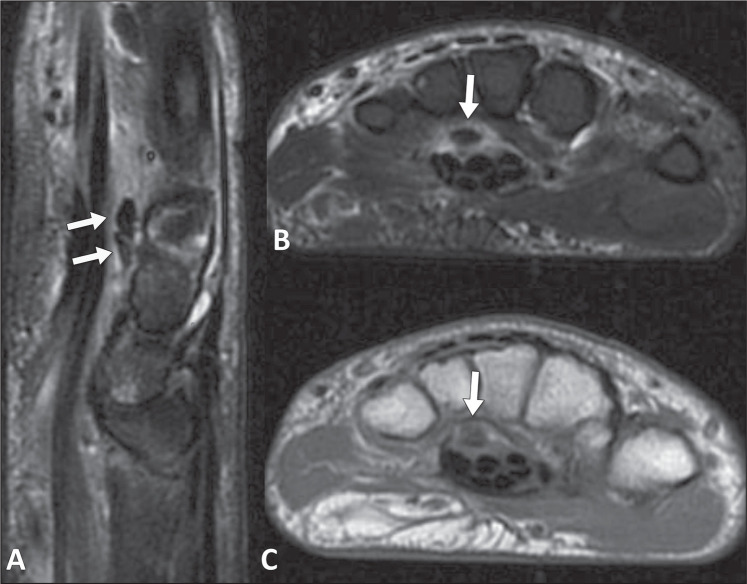
A 54-year-old male patient with pain and swelling in the wrist underwent magnetic resonance imaging for investigation of suspected septic arthritis. A: Sagittal T2-weighted image with fat saturation, showing low signal foci (arrows) in the carpal tunnel region, deep near the flexor tendons, with intense edema of adjacent soft tissues. B,C: Axial T2-weighted and T1-weighted images (B and C, respectively), showing low signal-intensity foci (arrows) within the carpal tunnel, with adjacent soft tissue edema extending to the flexor tendons, without proliferative synovitis or bone edema that would suggest inflammatory or septic arthritis.

Given the clinical presentation of ACP, infection is typically considered in the differential diagnosis. Symptoms with an acute onset, together with clear signs of local inflammation, can initially raise suspicion of septic arthritis (Figure 5). However, evidence of calcification on imaging studies makes infection unlikely, unless there is pre-existing calcification secondary to concomitant chronic kidney disease or crystalline arthropathy^(2)^. Although concomitant infection has been reported in the setting of ACP, it is a rare finding and should not be considered as the most likely possibility^(3)^.

**Figure 6:**
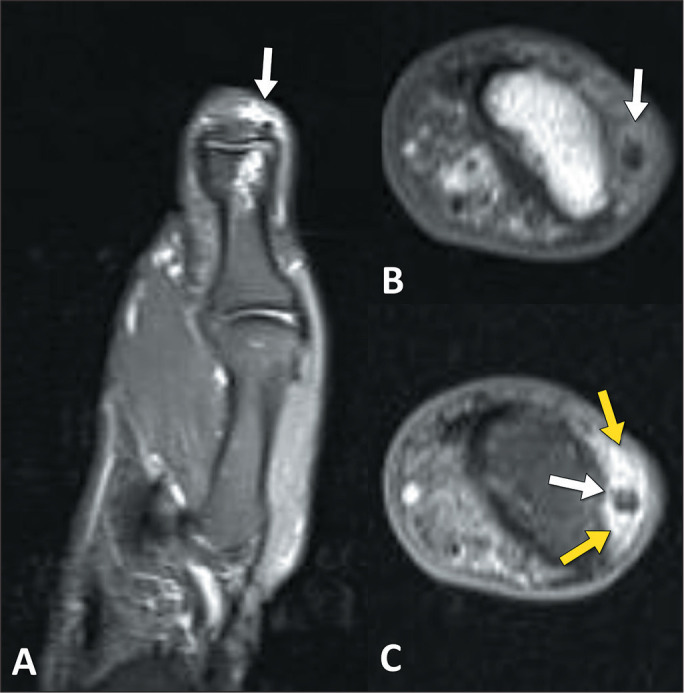
A 63-year-old male patient with pain in the thumb and suspected arthritis. A: Coronal T2-weighted image with fat saturation, showing a small focus of low signal intensity (arrow), with edema in the adjacent adipose planes. Note also the subchondral edema and cystic changes in the head of the proximal phalanx of the thumb. B,C: Unenhanced axial T1-weighted image (B) and contrast-enhanced T1-weighted image with fat saturation (C), showing a small focus of low signal intensity (white arrows) and enhancement in the adipose planes (yellow arrows), without proliferative synovitis that would suggest inflammatory or septic arthritis.

Magnetic resonance imaging is extremely useful for evaluating the site of calcium deposition, as well as the relationship with the surrounding structures, and for characterizing the local inflammatory process^(9)^, which makes it the method of choice to exclude the possibility of septic arthritis or inflammatory arthropathy of another nature. It should be borne in mind that small foci of calcific deposits can be missed on magnetic resonance imaging when no radiograph has been obtained or can be misinterpreted as accessory ossicles or avulsion fractures, particularly in the fingers and feet^(6)^, as depicted in Figure 6.

**Figure 7:**
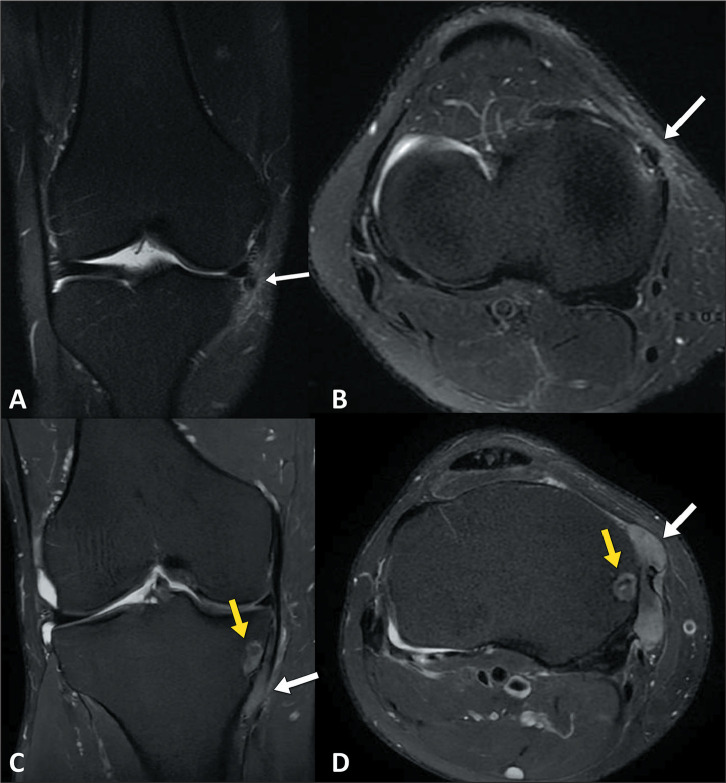
A,B: A 35-year-old male patient with pain in the medial aspect of the knee and suspected ligament injury. Coronal and axial T2-weighted images with fat saturation (A and B, respectively), revealing a focus of low signal intensity (arrows) in the medial capsular region of the knee, with edema of adjacent soft tissues. C,D: A 55-year-old male patient with a confirmed diagnosis of gout. Coronal and axial T2-weighted images with fat saturation (C and D, respectively), showing a soft tissue mass involving the medial collateral ligament, capsular structures, and pes anserine tendons, consistent with a gouty tophus (white arrows), with intraosseous extension (yellow arrows).The foci of urate crystal deposition (gouty tophi) present an intermediate signal, and the typical location, near ligaments and tendon structures, is a clue to distinguish gouty tophi from ACP.

Other diagnoses that can be radiologically differentiated from ACP are crystal arthropathies such as gout and calcium pyrophosphate dihydrate crystal deposition disease, because ACP is monoarticular and does not involve the joint itself. Unlike ACP, gouty arthritis manifests as periarticular masses with foci of calcification (gouty tophi) that can cause juxtacortical erosions, in a piecemeal pattern or invading the bone (Figure 7). Although periarticular calcifications may also occur in calcium pyrophosphate dihydrate crystal deposition disease, the presence of chondrocalcinosis is a useful distinguishing feature^(10)^.

Heterotopic ossification and accessory ossicles are distinguishable from ACP because they have a cortex and internal trabeculation^(2,10)^. Metastatic periarticular calcifications, which can result from stage 5 chronic kidney disease, hypoparathyroidism, tumoral calcinosis, vitamin D intoxication, or sarcoidosis, can mimic ACP calcifications, although the clinical presentation differs from that seen in those conditions^(9,10)^.

## CONCLUSION

Albeit uncommon, ACP is a major condition with marked symptoms and is often confused with other inflammatory joint diseases. Imaging methods are useful for characterizing calcific deposits. In particular, magnetic resonance imaging is important for identifying periarticular inflammatory changes related to ACP, as well as for excluding septic arthritis and inflammatory arthropathies of another nature, on the basis of the intra-articular inflammatory findings.
